# 

**Published:** 1916-01

**Authors:** 


### Contents

Event and Comment ........ 1

A System of Making Jacket Porcelain Crowns without Fusing... 7

The Treatment of Narrow Nasal Passages ........ 14

A Trip to the Panama-Pacific Dental Congress........ 16

Prevention—The Technique of the Future ........ 21

A Physician's Estimate of Modern Dentistry ........ 27

Indents ........ 31

Announcements ........ 45

Obituary ........ 48

